# Multi‐Mode Triboelectric Nanogenerator for Football Impact Monitoring and Mechanical Energy Harvesting

**DOI:** 10.1002/open.202400465

**Published:** 2025-03-29

**Authors:** Xi Chen, Xiaolong Yu

**Affiliations:** ^1^ Xuchang Vocational Technical College Xuchang 46 1000 China; ^2^ Physical Education School Shenzhen University Shenzhen 518060 China

**Keywords:** Triboelectric nanogenerators (TENGs), Dual-working mode, Football monitoring, Digital intelligence movement

## Abstract

The integration of digital products and sensors significantly enhances motion monitoring accuracy, addressing the limitations of hawk‐eye technology. Triboelectric nanogenerators (TENGs) provide innovative, low‐cost solutions for developing intelligent digital motion monitoring systems. In this study, we designed a multi‐mode triboelectric nanogenerator (M‐TENG) that incorporates multiple working modes, enabling switching between internal electrodes to adjust the output mode. This dual working mode enhances the adaptability of the device for football monitoring applications. The versatile design allows the M‐TENG to perform both energy harvesting and trigger‐based sensing and monitoring, contributing to the advancement of digital intelligence movement technologies. After optimization, the M‐TENG achieved a transferred charge (Qsc) of 88.38 nC, short‐circuit current (Isc) of 8.58 μA, and open‐circuit voltage (Voc) of 85.91 V, showcasing excellent electrical performance. The device charged a 1 μF capacitor to 5 V in 36 seconds, delivering a peak power of 178 μW, and maintained stable output with only a 14 % decrease over 60 days. Additionally, the M‐TENG effectively detects and harvests energy from football impacts, generating consistent voltage signals from each interaction, making it a promising candidate for real‐time sports equipment monitoring without the need for an external power source.

## Graphical abstract

A multi‐mode triboelectric nanogenerator (M‐TENG) is developed to enhance football monitoring by integrating energy harvesting and trigger‐based sensing. With switchable internal electrodes, it achieves optimized electrical performance (Qsc: 88.38 nC, Isc: 8.58 μA, Voc: 85.91 V) and stable output over 60 days. The M‐TENG offers a self‐powered solution for real‐time motion monitoring in digital sports technologies.

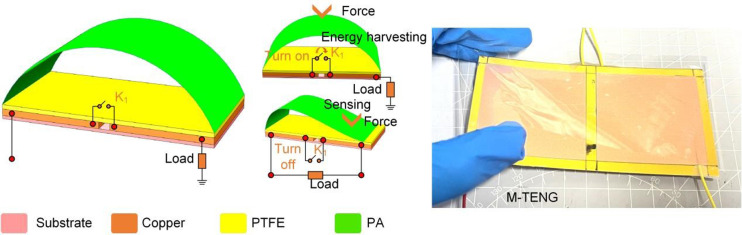

## 1 Introduction

In more recent years, with the progress of science technology, digital sports[^1^, ^2^] have become a new darling in the field of sports. Scientists have developed a variety of smart devices such as sports bracelets, sports trackers, smart courts,[^3^, ^4^, ^5^] etc, which can not only help athletes provide scientific training programs, but also provide a more intuitive and fair environment for sports events. However, many smart products have shortcomings such as difficult power supply,[^6^, ^7^, ^8^] weak sustainability,[^9^, ^10^] and cumbersome maintenance.[^11^, ^12^] Although the improvement of battery performance[^13^, ^14^] has further alleviated the energy storage[^15^, ^16^] and power supply problems,[^17^, ^18^] the power supply dependence of a variety of digital products and sensors still limits the development of intelligent motion monitoring.[^19^, ^20^, ^21^, ^22^, ^23^] Therefore, there is an urgent need to develop low‐carbon,^[24]^ energy‐saving^[25]^ and sustainable energy sources^[26]^ to power digital products, such as various self‐powered network sensors.[^27^, ^28^, ^29^, ^30^]

Triboelectric nanogenerator (TENG) is a kind of micropower collector, which was first proposed by Academician Wang Zhonglin's team in 2012.[^31^, ^32^] It uses mechanical energy in the environment to drive the friction motion of the generator. Due to electrocontact and electrostatic induction,[^33^, ^34^, ^35^] electrons will periodically pass through the load driven by potential difference, and then provide electrical energy to the load.[^36^, ^37^] Therefore, it can be used to power digital motion monitoring products. The TENG has four main working modes, namely, contact separation mode, horizontal sliding mode, single electrode mode and independent layer mode.[^38^, ^39^] It relies on common mechanical energy in life as a driver, such as body movement energy,[^40^, ^41^] vibration energy,[^42^, ^43^] wind energy,[^44^, ^45^] sound wave energy,[^46^, ^47^] ocean energy,[^48^, ^49^] droplet energy,[^50^, ^51^] etc. Thanks to the advantages of broad structural design, low‐priced and high applicability, TENG has developed rapidly in the fields of micro‐nano energy,^[52]^ self‐driven sensing, Marine blue energy,^[53]^ high‐voltage power supply,^[54]^ biomedicine^[55]^ in recent years, and can be applied in many fields such as self‐driven electrochemistry, human‐computer interaction,^[56]^ biomedical sensing,^[57]^ environmental and mobile sensing. TENG is also commonly used for athlete body monitoring, such as human footsteps monitoring,^[58]^ muscle stretching monitoring,^[59]^ heartbeat pulse monitoring,^[60]^ etc. It can also be installed in sports equipment or venues, such as landing point monitoring on table tennis table, displacement and speed monitoring on track and field, and water temperature monitoring in swimming gyms.[^61^, ^62^] Recent advances in computational methodologies, particularly those leveraging artificial intelligence (AI), have significantly accelerated the discovery of novel materials and the optimization of energy‐harvesting devices. AI‐driven approaches, such as machine learning models and high‐throughput simulations, have been successfully applied to predict triboelectric material performance, optimize device structures, and enhance energy conversion efficiency.[^63^, ^64^] In addition to experimental advancements, AI‐powered material design strategies enable the rapid screening of high‐performance triboelectric pairs, reducing the reliance on trial‐and‐error fabrication. Furthermore, deep learning‐based computational frameworks have been explored to model the charge transfer mechanisms in TENGs, paving the way for next‐generation self‐powered devices with higher efficiency and durability.^[65]^ These emerging computational methodologies provide a promising future direction for improving triboelectric energy harvesting systems and broadening their application in real‐world scenarios.

Here, we have developed a multi‐mode triboelectric nanogenerator (M‐TENG) capable of integrating various working modes by switching between internal electrodes, enabling both energy harvesting and trigger‐based sensing. Unlike conventional single‐mode TENGs, our design enhances adaptability for applications such as sports monitoring, particularly in football impact detection. The optimized structure and material selection ensure efficient performance, making it a promising candidate for self‐powered sensing systems. This work contributes to the advancement of intelligent motion monitoring by offering a sustainable, power‐independent solution for real‐time sports data collection. Unlike conventional TENGs that operate in a single mode, the proposed M‐TENG integrates multiple working configurations, allowing for switchable energy harvesting and sensing functions within a single device. This adaptability is particularly beneficial for real‐time motion monitoring, as it enhances sensitivity to different types of mechanical stimuli. Compared to existing impact‐based TENGs, which are often limited by fixed working modes and lower output stability, the M‐TENG introduces an internal electrode switching mechanism to optimize performance for varying impact conditions. Furthermore, while electromagnetic and piezoelectric harvesters are commonly used in motion sensing, they require complex fabrication and external power sources, whereas the M‐TENG provides a lightweight, cost‐effective, and fully self‐powered solution for football impact monitoring. These innovations distinguish our approach from previous TENG designs and enhance its potential for practical deployment in sports technology and intelligent motion sensing applications.

## 2 Results and Discussion

**Figure 1:**
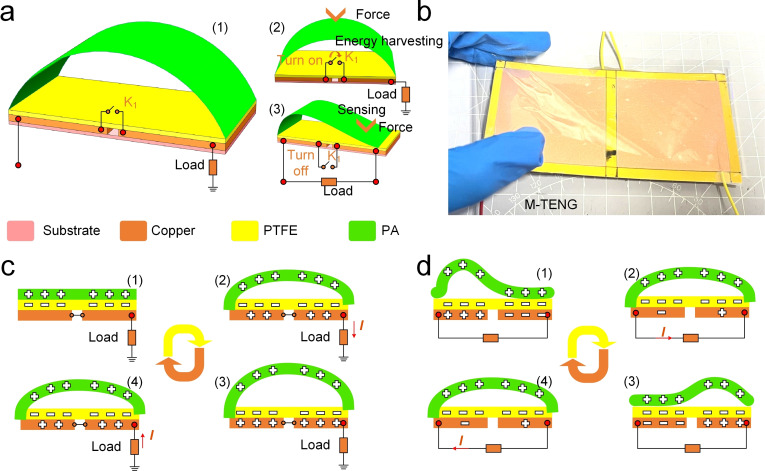
(a) Schematic illustration of the working principle of the M‐TENG, showing its dual functionality in energy harvesting and sensing states through triboelectric charge transfer and electrostatic induction. (b) Photograph of the fabricated M‐TENG prototype, highlighting its compact design and flexible structure suitable for practical applications. (c) Energy harvesting process of the M‐TENG, illustrating the sequential charge generation and transfer mechanism when subjected to mechanical impacts. (d) Sensing mechanism of the M‐TENG, demonstrating how the device detects external forces and converts them into distinguishable electrical signals for real‐time monitoring.

Figure 1(a1) illustrates the structural design and operational mechanism of the M‐TENG. The M‐TENG device comprises a substrate, two copper electrode, a PTFE dielectric layer, and a PA layer, arranged in an arch‐like configuration. The PTFE and PA layers are responsible for the triboelectric effect. This M‐TENG design incorporates dual working modes: one for mechanical energy harvesting and the other for triggered sensing and monitoring, facilitated by the integration of switches and output terminals. Figure 1(a2) demonstrates that when the switch is closed, the two conductive copper electrodes of the M‐TENG are connected, enabling the M‐TENG device to operate in single‐electrode mode for energy harvesting. When the switch is turned off, the M‐TENG device enters a sensing state by activating the specific PA film region located above the two copper electrodes, as illustrated in Figure 1(a3). Figure 1(b) presents a photograph of the fabricated M‐TENG prototype, where the device is shown in its assembled state, encapsulated in protective material. The image demonstrates the practical implementation of the M‐TENG, confirming its flexible design suitable for real‐world applications. Figure 1(c) illustrates the energy harvesting process, which operates based on the coupling of contact electrification and electrostatic induction. The M‐TENG consists of a PA layer and a PTFE layer. As shown in the sequence (1) to (4), when external mechanical force causes the PA layer to deform and come into contact with the PTFE layer, electrons are transferred between the two materials due to their different electron affinities. In the initial state (1), the PA and PTFE layers are separated, creating a positive charge on the PA and a negative charge on the PTFE. As deformation progresses (2), the PA layer moves downward, increasing the contact area, thereby facilitating the triboelectric charge transfer. The induced potential difference drives electrons to flow through the external circuit (3). Upon release of the applied force (4), the PA layer separates from the PTFE, restoring the charge imbalance, and generating a continuous alternating current as the process repeats. This cycle of deformation and recovery ensures a consistent energy output, which can be harvested to power external loads. The use of soft materials and flexible structures allows the M‐TENG to adapt to various mechanical inputs, making it highly effective for harvesting low‐frequency mechanical energy. When the PA film on the right side makes contact with the PTFE film in the corresponding area below, the surface charges on both the PA and PTFE films reach equilibrium, leading to a redistribution of charges between the two conductive copper electrodes, as shown in Figure 1(d1). Upon separation of the right PA film from the PTFE, the copper electrode beneath the right PTFE film induces charges of opposite polarity, generating a current signal in the circuit, as illustrated in Figure 1(d2). Similarly, when the left PA film contacts the PTFE film, the surface charge of the PTFE is neutralized, causing the copper electrode on the lower right side to induce a positive charge, which generates a current in the circuit, as illustrated in Figure 1(d3). As the left PA film detaches from the PTFE, the copper electrode induces a positive charge to balance the negative charge on the surface of the PTFE film, thereby contributing to the circuit's current flow, as illustrated in Figure 1(d4). The dual functionality of the M‐TENG as both an energy harvester and a pressure sensor makes it highly versatile for self‐powered sensing applications. By utilizing the triboelectric effect, the device can efficiently convert mechanical stimuli into electrical signals, enabling real‐time monitoring of environmental forces while simultaneously generating power for the system. This combination of energy harvesting and sensing functionalities positions the M‐TENG as a promising candidate for integration into self‐powered systems, particularly in scenarios where low‐power, autonomous operation is required.

**Figure 2:**
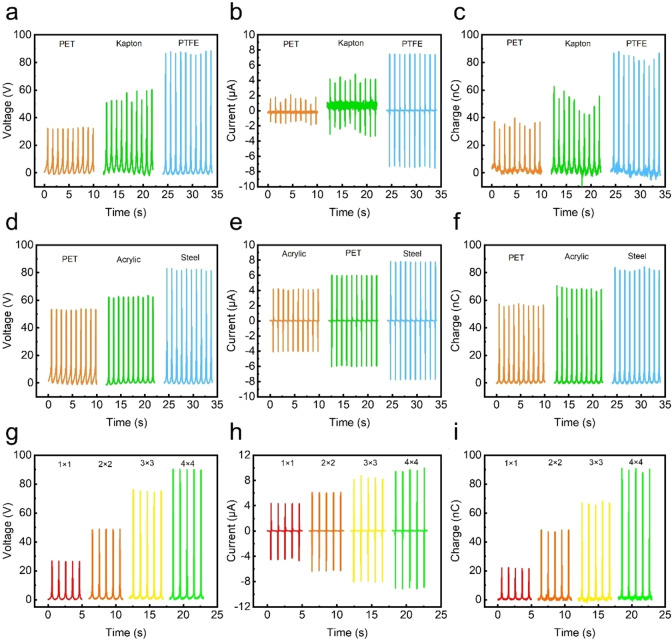
Electrical output performance of the M‐TENG under different structural configurations. (a) Voc, (b) Isc, and (c) Qsc of the M‐TENG using different negative triboelectric materials, demonstrating the impact of material selection on electrical performance. (d) Voc, (e) Isc, and (f) Qsc of the M‐TENG with different arch base materials, highlighting the influence of structural support materials on charge generation. (g) Voc, (h) Isc, and (i) Qsc of the M‐TENG with varying triboelectric layer sizes, showing the effect of active surface area on output performance.

Figure 2(a) shows the voltage output for each material over time, indicating distinct voltage peaks for each dielectric. PET exhibits the lowest voltage output, while PTFE generates the highest, suggesting that the triboelectric series plays a critical role in determining the overall voltage. Figure 2(b) illustrates the current output, where similar trends are observed, with PTFE producing the highest peak current and PET the lowest. Figure 2(c) presents the charge transfer between the layers, again showing that PTFE outperforms the other materials, confirming its superior triboelectric properties. Figure 2(d) shows that steel yields the highest voltage output, followed by acrylic and PET. Figure 2(e) further demonstrates the same trend in current output, where steel produces a significantly higher current than the other two materials. Figure 2(f) presents the charge distribution, where the steel configuration exhibits the highest charge transfer, followed by acrylic and PET, highlighting the enhanced electrical performance when metal‐based materials are used in the M‐TENG. Figure 2(g) demonstrates the voltage output of each configuration, showing that as the number of units increases, the voltage output rises proportionally, with the 4×4 structure generating the highest output. Similarly, Figure 2(h) shows the current output increasing with the number of units, and Figure 2(i) illustrates the charge output, which also follows the same pattern of increasing charge transfer with larger structural configurations. In summary, this research illustrates that both the choice of dielectric material and the geometric configuration significantly impact the electrical output performance of the M‐TENG. PTFE and steel exhibit superior triboelectric performance compared to PET and acrylic, while larger structural configurations result in higher voltage, current, and charge outputs. These results provide valuable insights for optimizing M‐TENG design for specific applications, particularly in energy harvesting and self‐powered sensing technologies.

**Figure 3:**
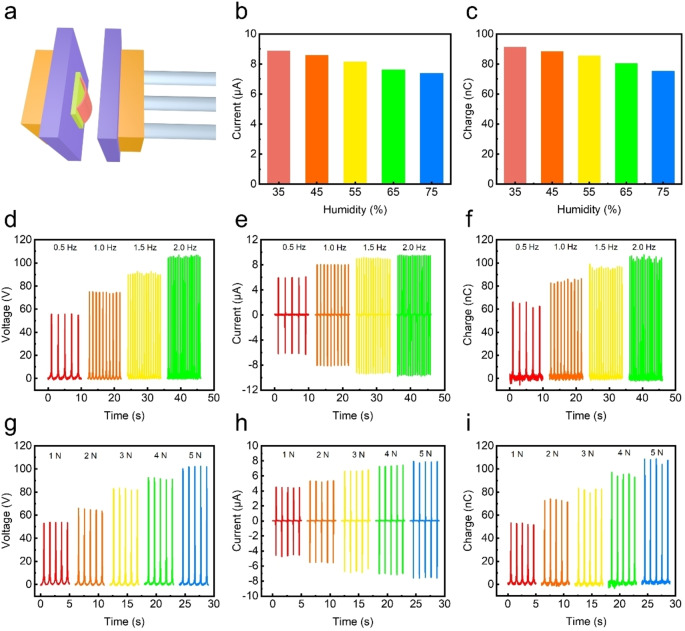
Electrical output performance of the M‐TENG under different external conditions. (a) Schematic diagram of the linear motor setup, used to provide controlled mechanical excitation for M‐TENG testing. (b) Isc and (c) Qsc of the M‐TENG under different humidity conditions, illustrating the effect of environmental moisture on electrical output. (d) Voc, (e) Isc, and (f) Qsc of the M‐TENG under varying motion frequencies, demonstrating the influence of excitation frequency on output performance. (g) Voc, (h) Isc, and (i) Qsc of the M‐TENG under different impact forces, showing the correlation between applied mechanical force and electrical response.

Figure 3(a) shows the linear motor model used in the parametric quantitative experiment of M‐TENG. Figure 3(b) and Figure 3(c) show the change of Isc and Qsc under different ambient humidity. It can be shown that when environment humidity increases from 35 % to 75 %, the impact on output of M‐TENG is mild, and the Isc and Qsc decrease slightly, which indicates that M‐TENG is not sensitive to ambient humidity and has good moisture resistance. Figure 3(d–f) shows the Voc, Isc and Qsc of M‐TENG at different moving frequencies. It can be observed that as the motion frequency gradually increases from 0.5 Hz to 2 Hz, the Voc increases from 57 V to 105 V, the Isc increases slightly from 6 μA to 9 μA, and the Qsc increases from 60 nC to 90 nC. This shows that the motion frequency has a great influence on the output. Figure 3(g–i) shows the influence of intensity on the output of M‐TENG. It is obvious that the impact strength gradually increases from 1 N to 5 N, and the Voc, Isc and Qsc all increase regularly. The increase of Isc is small, while the Voc and Qsc are doubled. It can be seen that within a reasonable impact force range, the increase of impact force will lead to a synchronous increase in output.

**Figure 4:**
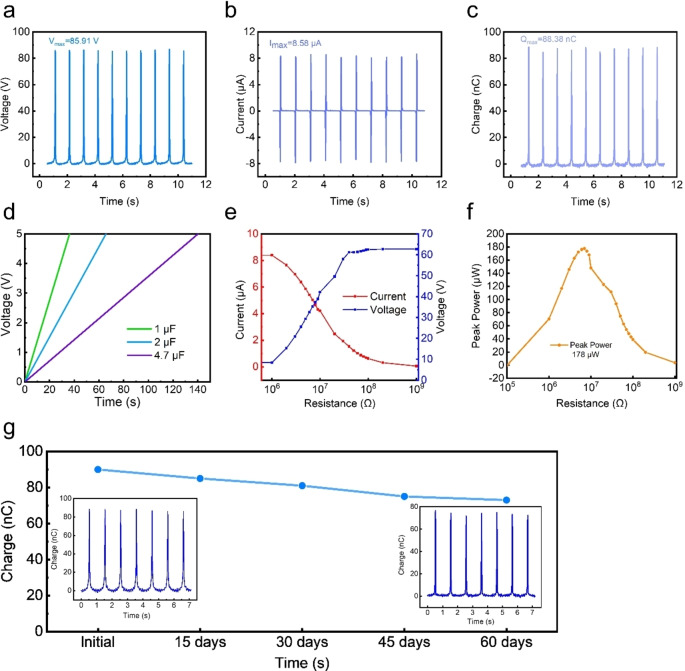
Electrical output characteristics and stability performance of the M‐TENG. (a) Voc, (b) Isc, and (c) Qsc at the optimized output state, demonstrating peak performance conditions. (d) Capacitor charging behavior of the M‐TENG, showing voltage buildup over time for different capacitance values. (e) Variation of output current and voltage under different external load resistances, illustrating load‐dependent electrical response. (f) Peak power output of the M‐TENG as a function of load resistance, identifying the optimal load for maximum power transfer. (g) Long‐term stability assessment, depicting the change in Qsc over 60 days to evaluate charge retention and durability.

As shown in Figure 4(a–c), the M‐TENG achieved a Qsc of 88.38±3.21 nC, Isc of 8.58±0.41 μA, and Voc of 85.91±2.75 V, based on five independent measurements. These results highlight the consistency and reliability of the device's performance. In addition, the application characteristics of M‐TENG also need to be explored. As shown in Figure 4(d), the charge capacity rate of the M‐TENG was measured. The M‐TENG device charged a 1 μF capacitor to 5 V in **36±2 s**, delivering a peak power of **178±5 μW**, ensuring reproducibility across multiple tests, as illustrated in Figure 4(e, f). Given that the active area of the M‐TENG is 25 cm^2^ (5 cm ×5 cm), the calculated power density is 7.12 μW/cm^2^. To further evaluate the advantages of the proposed M‐TENG, a comparative analysis was conducted against conventional energy harvesting technologies, including other TENG designs, piezoelectric nanogenerators (PENGs), and electromagnetic generators (EMGs). Compared to traditional TENGs, the M‐TENG exhibits a dual working mode, allowing for both energy harvesting and sensing functionalities. This versatility is rarely achieved in conventional TENG designs, which typically operate in a single mode. Additionally, the M‐TENG demonstrates an Voc of 85.91 V, which is higher than many previously reported impact‐based TENGs.[^66^, ^67^] In contrast, piezoelectric nanogenerators, while effective in vibrational energy harvesting, often exhibit lower voltage outputs and require complex fabrication processes. Furthermore, electromagnetic generators (EMGs) are commonly used for energy harvesting in dynamic environments but generally require bulky components and exhibit lower efficiency in small‐scale applications. The M‐TENG's lightweight structure and cost‐effective materials make it a promising alternative for real‐time motion monitoring and sports applications. However, one area for improvement is the energy conversion efficiency, which remains a challenge for triboelectric nanogenerators. Future work could focus on enhancing surface charge density through advanced materials or nanostructuring techniques to further boost output performance. These insights highlight the practical advantages of M‐TENG while identifying opportunities for future development. Figure 4(g) shows the output results of the M‐TENG at the beginning and after 60 days of placement. The long‐term stability of the M‐TENG was evaluated over a period of 60 days under controlled environmental conditions. The device was stored at a constant temperature of 25±2 °C and a relative humidity of 50±5 % in a standard laboratory environment. Performance measurements, including Qsc, Isc, and Voc, were recorded at 10 day intervals to monitor changes over time. After 60 days, the M‐TENG exhibited a performance degradation of approximately 14 %, confirming its durability under ambient conditions. These results demonstrate the device's potential for long‐term operation in practical applications.

**Figure 5:**
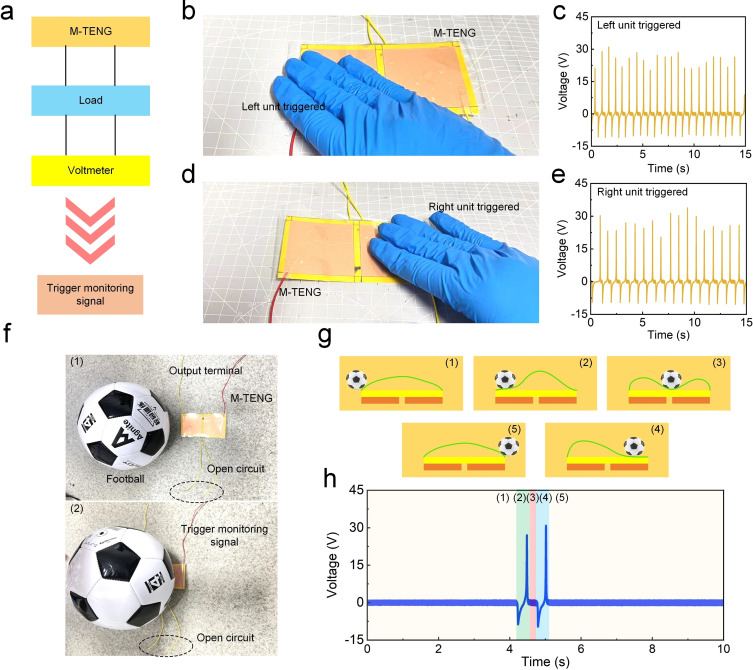
Demonstration of M‐TENG operation under applied pressure and football impact. (a) Schematic diagram of the M‐TENG circuit setup used for voltage signal monitoring during activation. (b) Activation of the left sensing unit of the M‐TENG under applied pressure. (c) Voltage output response corresponding to the left unit activation, showing real‐time signal generation. (d) Activation of the right sensing unit under applied pressure. (e) Voltage output response for right unit activation, illustrating symmetry in sensing functionality. (f1, f2) Demonstration of M‐TENG triggered by football impact, showcasing its ability to detect dynamic forces. (g1–g5) Schematic illustration of the M‐TENG's working mechanism during football impacts, detailing the charge transfer and sensing process. (h) Voltage output signals generated by football impacts, validating the M‐TENG's effectiveness in sports monitoring applications.

In Figure 5(a), a schematic of the circuit configuration is presented. The M‐TENG is connected to a load and a voltmeter, which monitors the voltage signals generated during the activation of the device. When the M‐TENG is triggered, it generates a signal that can be monitored for further analysis, demonstrating its utility as a self‐powered sensing device. Figures 5b and d show photographs of the experimental testing of the M‐TENG. In Figure 5(b), the left unit of the M‐TENG is triggered by pressing the corresponding section of the device, while Figure 5(d) depicts the right unit being activated under similar conditions. The pressure‐induced deformation of the PTFE layers within the device generates an electrical signal, which is recorded and further analyzed. This indicates that the M‐TENG can be used to detect directional force or pressure on different sections of the device, demonstrating its capability for spatially resolved pressure sensing. Figure 5(c, e) display the voltage signals corresponding to the activation of the left and right units, respectively. Both graphs reveal periodic voltage signals that fluctuate between approximately +30 V and −15 V, showing the ability of the M‐TENG to produce alternating current (AC) signals when subjected to repeated mechanical inputs. The output signals are highly consistent, with minimal noise, underscoring the M‐TENG's reliability in pressure sensing and its potential for continuous operation in real‐world applications. The amplitude and frequency of the signals correlate directly with the applied pressure, allowing for precise monitoring and quantification of external stimuli. Figure 5(f) showcases a practical demonstration of the M‐TENG's ability to detect and harvest energy from external impacts. In this case, a football is used as the mechanical trigger for the M‐TENG. Figure 5(f1) shows the initial setup, where the football is positioned above the M‐TENG, which is connected to an open circuit. Figure 5(f2) illustrates the output terminal of the M‐TENG as the football triggers the sensor, demonstrating the M‐TENG's ability to function as a self‐powered sensor in response to dynamic mechanical stimuli. In Figure 5(g), a detailed schematic outlines the step‐by‐step process of the M‐TENG's operation under the influence of the football. When the ball impacts the M‐TENG (1), it causes the PA film to come into contact with the PTFE layer beneath (2), generating triboelectric charges. As the football rolls away (3), the layers separate, leading to charge redistribution. The football continues to interact with the device through subsequent impacts (4) and (5), resulting in continuous energy harvesting and sensing. Finally, Figure 5(h) presents the voltage output corresponding to the impacts of the football on the M‐TENG, as shown in Figure 5g. The voltage signal is characterized by a series of sharp spikes, each corresponding to a distinct interaction between the football and the M‐TENG. The sequence of impacts, labeled (1) to (5), can be clearly identified in the voltage curve. Each spike in the voltage output indicates the contact and subsequent separation of the PA and PTFE layers, consistent with the triboelectric energy harvesting mechanism. The signal spikes vary in magnitude depending on the intensity and duration of the impacts, illustrating the M‐TENG's sensitivity to external mechanical forces. Overall, this application highlights the effectiveness of the M‐TENG as both an energy harvester and a self‐powered sensor. The device's ability to generate consistent voltage signals in response to mechanical stimuli, such as pressure or impact, demonstrates its potential for a wide range of applications, including sports equipment monitoring, autonomous sensing systems, and wearable technologies. The M‐TENG's dual functionality and flexibility make it a promising candidate for real‐world implementations in energy harvesting and environmental sensing.

## Experimental Section

### Materials

PTFE film, Polyamide (PA) film, spring steel sheet, acrylic base, PET sheet, Kapton tape, Kapton film, copper tape, dupont wire.

*Preparation of M‐TENG*: The M‐TENG device was fabricated using a flexible polyethylene terephthalate (PET) substrate (thickness: 125 μm). The copper electrodes (thickness: 50 μm) were patterned using a photolithography process with a positive photoresist (AZ5214E), developed at 23 °C for 60 seconds, followed by an etching process using ferric chloride (FeCl₃) solution at 40 °C for 5 minutes to define the electrode structures.

For the triboelectric layers, polyamide (PA) film (thickness: 50 μm) and polytetrafluoroethylene (PTFE) film (thickness: 30 μm) were selected due to their high triboelectric polarity. These layers were plasma‐treated at 50 W for 90 seconds to enhance surface charge retention and were laminated onto the copper electrodes using a pressure of 2 MPa at 60 °C for 10 minutes to ensure strong adhesion.

To create a multi‐mode configuration, a microswitch was integrated into the circuit, allowing mode transitions by adjusting the internal electrode connections. The entire assembly was encapsulated with a Kapton insulation layer (thickness: 25 μm), applied at 100 °C for 5 minutes, providing mechanical protection while maintaining the device's flexibility. The completed M‐TENG was tested under controlled conditions to evaluate its performance.

*Characterization and Measurements*: A homemade linear dynamic system (linear motor, controller and related programs) provides customizable mechanical motion for the M‐TENG. The Voc, Isc, and Qsc of M‐TENG were determined by electrometer (Keithley 6514).

## 3 Safety Assessment

Ensuring the safety of M‐TENG operation is critical, especially given its high voltage output. The maximum open‐circuit voltage (Voc) recorded was 85.91 V, which remains well below hazardous levels for human safety, as voltages below 100 V in dry conditions are generally considered safe according to IEC 60479–1 safety standards. Additionally, the short‐circuit current (Isc) of 8.58 μA is far below the threshold for electrical hazards, confirming that the device does not pose a risk of electric shock.

To enhance safety, a Kapton insulation layer (thickness: 25 μm) was applied to prevent unintentional contact with conductive components. The M‐TENG operates in a self‐powered manner, eliminating the risks associated with external power supplies or batteries. Moreover, thermal stability was assessed by exposing the device to 50 °C for 24 hours, with no significant degradation in electrical performance. These measures ensure that the M‐TENG is safe for prolonged use in various applications, including sports monitoring and wearable electronics.

## 4 Cost‐Benefit Analysis

One of the significant advantages of the M‐TENG is its low‐cost fabrication, which makes it a promising alternative to conventional energy harvesters. The primary materials used in its construction, including PET film, PTFE film, and copper electrodes, are widely available and inexpensive compared to the rare‐earth materials used in electromagnetic and piezoelectric harvesters. The estimated cost per device is under $5, making it feasible for mass production.

Additionally, the fabrication process involves simple steps such as screen printing, lamination, and basic electrode assembly, eliminating the need for complex microfabrication techniques. This not only reduces production costs but also makes the M‐TENG compatible with scalable roll‐to‐roll manufacturing methods, enhancing its potential for commercialization.

From a performance perspective, the M‐TENG achieves a power density of 7.12 μW/cm^2^, which is comparable to or higher than many existing TENG designs. Given its low production cost, ease of manufacturing, and strong performance metrics, M‐TENG offers a cost‐effective solution for self‐powered sensing applications, particularly in sports monitoring, wearable electronics, and IoT devices. These attributes make it a compelling candidate for real‐world deployment, bridging the gap between research innovation and commercial feasibility.

## 5 Conclusions

In conclusion, we successfully designed a multi‐mode M‐TENG with the capability to switch between internal electrodes, allowing for flexible output modes. This design enables the M‐TENG to function in both energy harvesting and trigger‐based sensing modes. Following optimization, the M‐TENG achieved excellent electrical performance, with a transferred charge (Qsc) of 88.38 nC, short‐circuit current (Isc) of 8.58 μA, and open‐circuit voltage (Voc) of 85.91 V. The device charged a 1 μF capacitor to 5 V within 36 seconds, with a peak power output of 178 μW, and demonstrated long‐term stability, with only a 14 % output decrease over 60 days. Moreover, the M‐TENG effectively detected and harvested energy from football impacts, generating consistent voltage signals with each interaction. This highlights its potential as a self‐powered solution for real‐time sports equipment monitoring, eliminating the need for external power sources.

## Conflict of Interests

The authors declare that they have no conflict of interest.

## Data Availability

The data that support the findings of this study are available from the corresponding author upon reasonable request.
